# Prostate cancer treatment with electrochemotherapy (ECT): safety, efficacy and clinical experience in 144 patients

**DOI:** 10.2478/raon-2025-0061

**Published:** 2025-12-16

**Authors:** Mara Stevanovic, Mathias Heringer, Mohammad Hjouj, Alessandro Zanasi, Francesca de Terlizzi, Michael K Stehling

**Affiliations:** 1 VITUS Privatklinik, Offenbach, Germany; 2 Department of Medical Imaging, Al-Quds University, Jerusalem; 3 IGEA S.p.a. Biophysics Lab. Carpi, Modena, Italy

**Keywords:** prostate cancer, focal therapy, electrochemotherapy, local therapy, local progression free survival

## Abstract

**Background:**

Prostate cancer (PCa) is a common cancer in men in developed countries. PCa treatment depends on the disease’s stage; focal therapy provides an intermediate approach, with lower toxicity compared to radical prostatectomy, and better tumor control than active surveillance. We report the first retrospective analysis of prostate cancer patients treated with ECT at our institution.

**Patients and methods:**

A cohort of 144 men with prostate cancer who were ineligible for or refused standard therapies were included and treated with ECT. Routine follow-up included PSA tests and MRI scans, as well as toxicity and genitourinary function evaluation by means of standard questionnaires. Local response was evaluated with MRI at 3 months after treatment, following the RECIST criteria for solid tumors.

**Results:**

The procedure was technically successful in all patients and was well tolerated, with mild and temporary adverse events. Urinary and erectile functions were mostly preserved. A complete response was observed in 75% of evaluated patients, a partial response in 18%, stable disease in 5%, and disease progression in 2%. Short-term response was associated with TNM stage (p < 0.05), Gleason score (p = 0.0066), and pre-ECT PSA levels (p = 0.0070). During follow-up, 18 patients (13%) experienced disease progression; 1-year PFS was 88% (95% CI: 80%–97%) and was found to be significantly associated with tumor stage and pre-treatment PSA levels.

**Conclusions:**

ECT is a feasible, safe, and effective treatment for prostate cancer, with extremely low toxicity and side effects. Preliminary results suggest that it offers promising outcomes in terms of local disease control in early-stage tumors, but also in locally advanced cases where other treatments may not be viable.

## Introduction

Prostate cancer (PCa) is the second most common non-skin cancer in men and a major cause of cancer-related mortality in developed countries. In 2022, PCa occurred in an estimated 1.5 million new cases and was responsible for approximately 397,000 deaths worldwide.^1^

Two well-established risk factors for prostate cancer are advanced age (over 65) and family history.^2^ While smoking, obesity, and certain dietary factors are speculated to influence prostate cancer risk, genetic susceptibility and Western African ancestry are also significant contributors.^3^

Prostate cancer treatment depends on the stage of the disease, with different approaches used for localized, locally advanced, and metastatic cases. For localized disease, current treatment options include active surveillance or watchful waiting for low-risk PCa, and radical prostatectomy (RPE) or radiation therapy (RT) for intermediate-risk and high-risk PCa.4 With a median PSA level of 15 ng/ml for prostate cancer treated with RPE and radiotherapy, there is a slight reduction in mortality, metastases, and local progression. At the same time, the incidence of adverse effects, such as urinary incontinence and erectile dysfunction, remains significant.^5^ Locally advanced prostate cancer, defined as PCa spreading outside the prostate but not metastasized, is typically treated with a combination of radiation and androgen deprivation therapy (ADT).4 In contrast, metastatic disease is treated primarily with ADT, sometimes combined with chemotherapy or androgen receptor-targeted therapies. However, resistance to treatment often results in castration-resistant prostate cancer (CRPC), which remains difficult to treat and has a poor prognosis.^6^

Focal therapy (FT), including high-intensity focused ultrasound (HIFU), cryotherapy, radiofrequency ablation (RFA), irreversible electroporation (IRE), and focal radiation, provide an intermediate and innovative approach to PCa^7^, offering lower toxicity compared to radical prostatectomy, while providing better tumor control than active surveillance.^7^ FT targets the tumor while sparing healthy tissue, offering a less invasive option with a lower risk of side effects such as incontinence and erectile dysfunction. Until now, FT has been limited to carefully selected patients with localized focal disease.8,9 Balancing treatment efficacy, extent of intervention, and quality of life remains a major challenge at all stages of the disease.

Electrochemotherapy (ECT) is a localized, nonthermal anticancer technique that combines reversible electroporation of tumor cells with concurrent, low-dose intravenous chemotherapy, usually bleomycin. Reversible electroporation enhances bleomycin efficacy by enabling it to reach its intracellular target.^10^ ECT has been established as an effective treatment for superficial tumors, including squamous cell carcinoma, basal cell carcinoma, sarcoma, and cutaneous metastases of various histological origin.^11^ Additionally, ECT has been applied to deep-seated tumors such as hepatocellular carcinoma, colorectal liver metastases, and bone metastases.^12–15^ ECT strategies for deep-seated tumors include open surgery, percutaneous, and laparoscopic techniques.^13,16,17^ The major advantage of ECT is its selective ability to kill dividing cancer cells while sparing non-dividing healthy cells and surrounding anatomical structures.^18^

To date, ECT in prostate cancer patients has been described in one case report only, involving locally advanced prostate cancer with infiltration of the external urethral sphincter, for which radical prostatectomy or radiation therapy would have most likely resulted in incontinence and impotence. ECT was effective with no sign of tumor activity on MRI at 6 months follow-up, whilst fully preserving sphincter function. The patient maintained continence and potency within the prior scoring range, indicating that ECT for prostate cancer is safe, feasible and can even be performed in patients with PCas which are not organ-confined.^19^ In this first large cohort study, we report the outcomes and safety profiles of prostate cancer patients treated with ECT to confirm its feasibility, efficacy, and impact on recurrence-free survival.

## Patients and methods

### Patients

Men with prostate cancer who were ineligible for or refused standard therapies were included. Inclusion criteria were: histological or radiological diagnosis of prostate cancer at any stage, age ≥18 years, Eastern Cooperative Oncology Group (ECOG) performance status ≤ 2, and life expectancy of at least three months. Exclusion criteria were: previous allergic reactions to bleomycin or any components required for anaesthesia, exceeding a cumulative lifetime dose of 250 mg bleomycin/m^2^ body surface (400,000 IU bleomycin/m^2^) previously exceeded, chronic renal dysfunction (serum creatinine > 150 μmol/L), or acute lung dysfunction.

All patients were informed about the nature of their disease, prognosis, standard treatment options according to the S3 guidelines for PCa issued by the German Urological Society^20^, the experimental nature of the ECT treatment, and details of the diagnostic work-up regarding tumor localization. Patients signed informed consent for treatment, which was personalized based on individual medical needs and preferences. Each treatment was personalized according to the patient’s individual needs and wishes (individual medical treatment). Data collection was purely retrospective. No treatment was adapted to suit scientific purposes. All procedures performed in present study were in accordance with the ethical standards and with the 1964 Helsinki declaration and its later amendments or comparable ethical standards.

### ECT treatment

All patients underwent multi-parametric magnetic resonance imaging (mpMRI) at least once prior to ECT. All but one patient had histopathological confirmation of PCa.

The procedure was performed with the patient in the lithotomy position, followed by disinfection and sterile covering of the pelvic floor area. Urethral gel was instilled, and catheterization (Charr. 16) with continuous bladder irrigation was performed. Transrectal ultrasound was used to visualize the insertion and final position of 5 to 8 electrodes (IGEA Spa, Carpi, Italy) depending on the size and shape of the lesion, guided by preoperative MRI images. The electrodes were manually inserted through the perineum under ultrasound guidance. Electrode positioning was planned based on MRI images ensuring that electrode couples to be activated were optimally placed max. 3 cm apart and a needle geometry covering the target volume was obtained. The catheter was then retracted into the mid-urethra. The body-surface-adjusted bleomycin dose was administered intravenously in a bolus. Eight minutes after intravenous bleomycin administration, reversible electroporation was performed by applying between 8 and 24 electric pulses of 1000 V/cm electric field intensity per electrodes pair, with possible repositioning of electrodes at varying depths within the organ to fully cover the lesion. After electrode retraction, the bladder was re-catheterized with a Charr. 16 transurethral catheter, and continuous bladder irrigation was resumed. Periprostatic local anaesthesia was administered with ropivacaine (20–40 mg), and peri-interventional antibiotic prophylaxis was performed. The whole procedure was performed under general anaesthesia, and patients were expected to stay in the hospital for 2 nights: admitted the day before treatment, they underwent preparation with laxatives and antibiotic prophylaxis; after treatment, they stayed overnight for observation and discharged the next day. All treatments were performed using the Cliniporator® (IGEA Spa, Italy).

### Follow-up

Routine follow-up included PSA tests and MRI scans, following protocols suggested for other local treatments.^21^ PSA testing was recommended every 3 months during the first 2 years, then every 6 months. MRI was performed 1 day after treatment, and at 3 and 9 months, then annually. Biochemical recurrence was defined as a rise in PSA above baseline 3 months post-treatment, confirmed by mpMRI and, if necessary, additional biopsy or prostate-specific membrane antigen (PSMA)-PET/CT. Patients were also interviewed regarding adverse events and their evolution over time.

### Safety and toxicity

Acute toxicity was recorded intra- and post-operatively until the removal of the bladder catheter. All patients had an MRI 24 hours post-ECT to confirm the alignment of the treatment field with the tumor extent and to assess any procedure-related side effects, such as haemorrhage or rectal damage.

### Assessment of genitourinary function

All patients were asked to complete the International Prostate Symptom Score (IPSS) questionnaire to evaluate urinary toxicity before and after ECT. The International Consultation on Incontinence Questionnaire-Urinary Incontinence (ICIQ-UI) was also used to assess continence status pre- and post-treatment and during follow-up. Erectile function was evaluated using the standard International Index of Erectile Function (IIEF-5) score before and after ECT.

### Response evaluation

Local response was evaluated with MRI at 3 months after treatment, following the RECIST criteria for solid tumors: complete response (CR) was defined as disappearance of the target lesion; partial response (PR) with at least 30% decrease in the size of the target lesion. Progressive disease (PD) was defined as at least 20% increase in the size of the target lesion and stable disease (SD) with neither sufficient shrinkage to qualify for PR or sufficient increase to qualify for PD. MRI and PSA were used to evaluate treatment response over time. PSMA-PET/CT and re-biopsy were performed when either PSA or MRI indicated a new suspicious lesion. Biochemical recurrence was defined by three consecutive PSA increases (ASTRO definition).^22^ MRI recurrence was assessed based on PI-RADS v2 criteria.^23^ Cases with suspected recurrences were discussed by a multidisciplinary board of urologists, oncologists, and radiologists. Re-biopsy was recommended for confirmed or strongly suspected recurrences, with re-treatment considered based on the patient’s preference.

Since most patients declined re-biopsy, PSMA-PET/CT with Gallium 69 was used in addition to MRI for local re-staging and whole-body re-staging.

### Statistical analysis

Descriptive statistics, including mean, standard deviation, median, and range, were used for continuous variables. Categorical variables were described by absolute numbers and percentages. Chi-square tests and contingency table analysis were used for comparisons of categorical variables. The Wilcoxon-Mann-Whitney test assessed differences in paired continuous variables (e.g., questionnaire outcomes). Kaplan-Meier curves were calculated for progression-free survival (PFS) for the entire cohort and subgroups, with statistical significance set at p < 0.05. NCSS 9 software (NCSS, LLC. Kaysville, Utah, USA) was used for statistical analysis.

## Results

A cohort of 144 patients with prostate cancer was treated with electrochemotherapy (ECT) at the VITUS Clinic Institute between January 2017 and June 2024. The baseline characteristics of the patients are presented in Table 1.

**TABLE 1.** Descriptive characteristics of patients included

	Mean	St.Dev.	Median	Min	Max
Age (yrs)	68	8	67	50	83
Height (cm)	180	7	180	156	196
Weight (kg)	81	11	82	51	110
PSA (ng/ml)	24.9	28.5	15.1	1.8	177.0
Prostate volume (ml)	44	18	40	6	117

Cancer stage and grade of the cohort are described in Table 2. Patients with Gleason Score of 6 or 7a (3+4) have been specifically analysed as they represent a low-intermediate-risk class of patients: 52 patients with mean age 67±7 yrs, mean PSA 16.8±10.4 ng/ml. Patients that received previous treatments, as reported in Table 3.

**TABLE 2.** Patient cancer stage and grade

Gleason score	N	%
6 (3+3) + 7a (3+4)	52	36%
7b (4+3)	40	28%
8–10 (4+4/5+3/4+5/5+4/5+5)	51	35%
Not available (no biopsy)	1	1%
**D’Amico risk classification**		
Low	2	1%
Intermediate	18	13%
High	124	86%
**Stage**	N	%
T1c	6	4%
T2a	16	11%
T2b	3	2%
T2c	25	18%
T3a	36	25%
T3b	26	18%
T4	32	22%
N0	111	77%
N1	33	23%
M0	112	78%
M1	32	22%

**TABLE 3.** Previous treatments

Previous treatments		
No	95	66%
Irreversible electroporation (IRE)	10	7%
Systemic therapy	8	6%
Radiotherapy/Radioligand	5	4%
Radical prostatectomy/TURP	3	2%
High-intensity focused ultrasound (HIFU)	2	1%
Thermoablation	2	1%
Multiple	19	13%

1 TURP = transurethral resection of the prostate

### Treatment

The procedure was technically successful in all patients. Treatment coverage included the entire prostate, with tumor volume extending beyond the capsule in 133 patients (93%), half of the organ in 6 cases (4%), focal treatment in 3 patients (2%), whilst treatment was palliative without complete tumor volume coverage in 2 patients (1%). The mean duration of the procedure was 106 ± 26 minutes (time in the operating theatre), and the mean intravenous bleomycin dose administered was 29 ± 1 mg. The mean number of variable geometry electrodes used during each treatment was 7 ± 1, ranging from 5 to 8 electrodes.

### Toxicity

The treatment was well tolerated, with adverse events being mild and temporary. These were primarily characterized by intraprostatic oedema in 21 patients (14.5%) and anterior rectal wall irritation in 5 patients (3.4%).

### Quality of life (QoL)

A total of 86 patients completed QoL questionnaires (ICIQ, IPSS, IIEF-5) before ECT, and 60 completed them after ECT. Data from pre- and post-ECT questionnaires are available for 36 patients.

### Urinary continence

In patients who were fully continent before ECT, no urinary incontinence was observed during the follow-up period. Nine percent of patients reported severe incontinence (ICIQ > 10) prior to ECT; this percentage remained similar (10%) during the first year of follow-up and dropped to zero after 12 months.

In patients Gleason Score 6 or 7a, only 1 patient out of 52 (2%) reported severe incontinence (ICIQ > 10) prior to ECT; this percentage slightly increased (6%) during the first year of follow-up and dropped to zero after 12 months.

Similar results were observed with the IPSS score, where 5% of patients reported severe urinary incontinence (IPSS ≥ 20) before ECT. This increased slightly to 6% during the first 12 months of follow-up but dropped to 0% after one year.

In patients at low-intermediate-risk, only 1 of the 52 patients (2%) reported severe urinary incontinence (IPSS ≥ 20) before ECT. At follow-up no patients in this group reported sever urinary incontinence.

### Erectile function

Nine patients (6%) developed severe erectile dysfunction after ECT, as indicated by a decrease in their IIEF-5 score from normal values before treatment, to a score ≤ 7 afterwards, which persisted for longer than one year in only one patient (0.7%).

**FIGURE 1.**
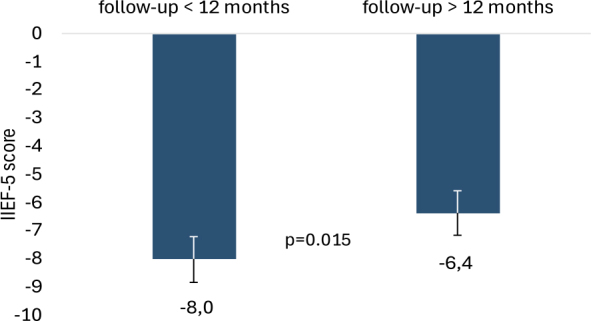
Mean International Index of Erectile Function (IIEF-5) reduction at follow-up with respect to pre-ECT values, during the first 12 months of follow-up and after 12 months of follow-up.

Overall, 56% of patients had impaired erectile function before ECT (defined as an IIEF-5 score < 22), of which 17% presented a severe erectile dysfunction. After treatment, a decrease of -8.0±9.4 is observed during the first year, which significantly reduces the gap to -6.4±8.0 after the first 12 months (p = 0.015) (Figure 1).

In the group of patients with Gleason Score of 6 or 7a, 10% suffered for severe erectile disfunction before ECT, but only 4% had impaired erectile function after 12 months from ECT.

### Short term response to ECT

**FIGURE 2.**
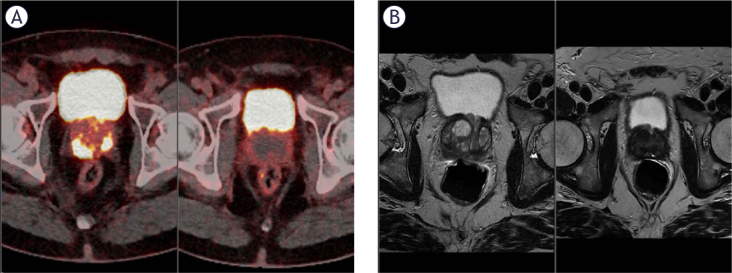
61 years old patient with biopsy-confirmed Gleason 6 multifocal prostate cancer but based on tracer uptake on prostate-specific membrane antigen (PSMA) PET and apparent diffusion coefficients (ADCs) on MRI, a higher-grade cancer. **(A)** Gallium-68 PSMA PET CT scan of the prostate before treatment (left) and 3 months after ECT (right). **(B)** MRI images of the prostate before treatment (left) and approximately 3 months after ECT (right).

Short-term response was evaluated at the 3-month follow-up and defined as a reduction in PSA levels, confirmed by a negative MRI performed within 1 month of the PSA evaluation. Short-term response data are available for 117 patients (81%). A complete response was observed in 88 patients (75%) (Figure 2), a partial response in 21 patients (18%), stable disease in 6 patients (5%), and disease progression in 2 patients (2%). Short-term response was associated with the TNM stage of the disease, the Gleason score, and pre-ECT PSA levels, as detailed in Table 4.

**TABLE 4.** Association between short term response and disease specific characteristics

SHORT TERM RESPONSE				P value
**T Stage**	**T1/T2**	**T3/T4**		
CR	92%	66%		
PR	5%	25%		
SD	3%	6%		
PD	0%	3%		0.0190
**N-M Stage**	**N0 and M0**	**N1 or M1**		
CR	89%	43%		
PR	9%	40%		
SD	1%	14%		
PD	1%	3%		< 0.0001
**Gleason score**	**6–7a**	**7b**	**8–10**	
CR	98%	68%	56%	
PR	0%	26%	32%	
SD	0%	3%	12%	
PD	2%	3%	0%	0.0002
**PSA level**	**< 10 ng/ml**	**> 10 ng/ml**		
CR	94%	66%		
PR	2%	26%		
SD	2%	7%		
PD	2%	1%		0.0070

1 CR = complete response; PD = progressive disease; PR = progressive disease; SD = stable disease)

### Long term response

During the follow-up period, 18 patients (13%) experienced disease progression. Among these, 11 patients (8%) had local progression or recurrence within the prostate, 5 (3%) developed systemic progression (involving lymph nodes, bones, or distant metastases), and further 2 patients (1%) experienced both conditions. The mean time to progression was 15 ± 6 months (median 16, range 3–25 months). Progression-free survival (PFS) was calculated based on both local and systemic disease progression, and the 1-year PFS for the entire cohort was 88% (95% CI: 80%–97%). This figure is similar when considering only local progression (1-year local progression-free survival [LPFS] of 92%, 95% CI: 84%–99%).

**FIGURE 3.**
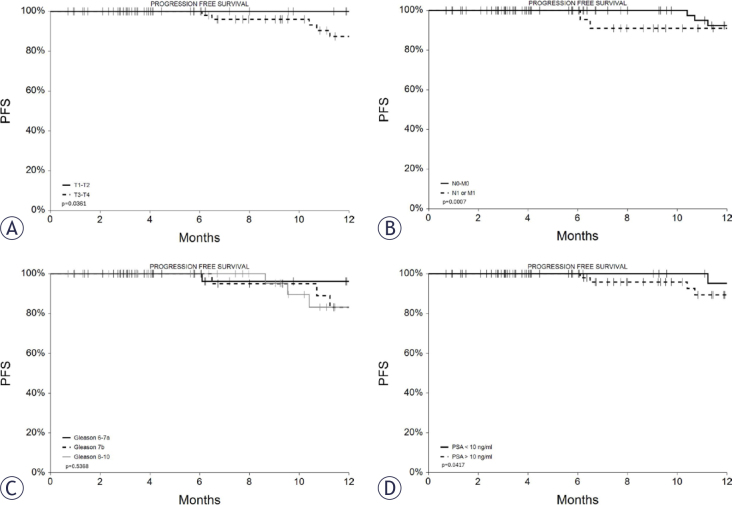
Progression free survival (PFS) curves for patients according to stage: **(A)** T1/T2 *vs*. T3/T4, **(B)** N0 and M0 *vs*. N1 or M1, **(C)** Gleason score, **(D)** PSA value

PFS was found to be directly and significantly associated with tumor stage and pre-treatment PSA levels, as shown in Figure 3 and Table 5. However, it was not related to the Gleason score.

**TABLE 5.** One-year progression free survival in the analysis by subgroups

GROUPS	1-year PFS	C.I. 95%	N at risk at 12 months	P value
**Whole cohort**	88%	80%–97%	43	
**Stage**				
T1/T2	100%	100%	15	
T3/T4	83%	71%–95%	28	0.0361
N0 and M0	92%	84%–100%	33	
N1 or M1	79%	61%–98%	10	0.0007
**Gleason score**				
6–7a	96%	89%–100%	19	
7b	83%	66%–100%	13	
8–10	83%	66%–100%	10	0.5368
**PSA**				
< 10 ng/ml	95%	86%–100%	20	
> 10 ng/ml	84%	72%–96%	23	0.0417

### Patients under concomitant immunotherapy

In this cohort of patients, 10 subjects who received ECT were undergoing immunotherapy (pembrolizumab). No increased toxicity was observed in these patients, as only two experienced mild, temporary oedema after the ECT treatment. Additionally, only one patient had a recurrence 9 months after the ECT session. This patient was a young individual (55 years old) with high-risk disease, a Gleason score of 9, T4N1M1 staging, and a PSA level of 90 ng/ml. No cases of urinary or erectile dysfunction were observed in any of these patients.

## Discussion

Focal therapy is an option for treatment of PCa with the aim of reducing known side effects of primary whole-gland treatments such as radical prostatectomy or radiotherapy and simultaneously providing sufficient oncological control. In the earlier days, FT was only considered for low-risk patients. During the past two decades however, there has been a gradual shift in FT towards targeting larger volumes and higher grades of PCa.^24–26^

Electrochemotherapy (ECT) is a minimally invasive, local tumour ablation technique that combines chemotherapy with transient permeabilization of the cell membrane by reversible electroporation. The latter is achieved by applying a series of short non-thermal electric pulses through electrodes that are placed directly at the site of the tumor. Potent chemotherapeutics which are poorly permeant under normal conditions can thus easily pass the membrane barrier and reach their intracellular target. Cytotoxicity is thereby dramatically increased: the effect of bleomycin, the most utilized drug for ECT, is potentiated several hundred times when combined with electroporation in vitro.^27^ At a preclinical level, a significant improvement of the anticancer activity of bleomycin via reversible electroporation was also shown in mice bearing human prostate cancer xenograft.^28^

In this study, to our knowledge, the first observational cohort study on the use of ECT in the treatment of prostate cancer, a cohort of 144 patients with prostate cancer at various stages underwent ECT. According to D’Amico Risk classification 86% of the patients had high-risk, 13% intermediate risk and only 1% low risk PCa. 34% of the patients had previously received other treatments, such as IRE, surgery, radiotherapy, or systemic therapies, but experienced recurrence or progression, with no further therapeutic options deemed feasible or indicated; 66% of the patients received ECT as a primary local treatment after having refused standard therapies and aiming to obtain better tumor control than active surveillance while reducing toxicity compared to radical treatments.

Most patients underwent a technically successful treatment, with ECT with coverage of the entire tumor site. Tumor volume extended beyond the prostate capsule in 133 patients (93%), involved half of the organ in 6 cases (4%), and was focal in 3 patients (2%). Only in 2 patients was the tumor so extensive that it could not be fully covered, resulting in palliative treatment.

The treatment was well tolerated, with only mild adverse events observed, such as intraprostatic oedema in 21 patients (14.5%) and anterior rectal wall irritation in 5 patients (3.4%). The major benefit of ECT lies in its selective capacity to destroy dividing cancer cells while preserving nondividing healthy cells and surrounding anatomical structures^10,18^, resulting in reduced toxicity and fewer side effects post-treatment.

Urinary and erectile functions were indeed preserved. Among patients with good urinary functionality prior to treatment, no urinary incontinence was observed post-ECT or during the follow-up period. In those who experienced some urinary incontinence before ECT, ranging from 5% to 9% depending on the evaluation questionnaire (ICIQ or IPSS), dysfunction persisted during the first year of follow-up but resolved completely after 12 months. This finding aligns with our previous study on patients who underwent irreversible electroporation (IRE)^9^ and is likely due to treatment-induced reduction of benign cellular hyperplasia in the prostate’s transitional zone, whilst non-cellular elements (primarily fibers) remained in place and temporarily obstructed urinary outflow. In contrast, standard treatments for prostate cancer have shown urinary incontinence rates ranging from 36% to 49% after radical prostatectomy^29^, and nocturia rates of 42% to 43% after external beam radiotherapy (eBRT) or brachytherapy (BT), with urinary incontinence occurring in 1% to 10% of cases.^29–31^

In this cohort, 9 patients (6%) developed erectile dysfunction post-ECT. Notably, 56% of patients already had impaired erectile function prior to treatment, with 17% experiencing severe erectile dysfunction. After 12 months of follow-up, erectile dysfunction persisted in only 1 of the 9 patients, while the condition resolved in the remaining 8. As anticipated, the rate of erectile dysfunction following ECT is significantly lower compared to other treatments: radical prostatectomy (80%–95%)^29^,^30^, eBRT (69%)^23^, high-intensity focused ultrasound (HIFU) (36%)^32^, cryosurgery (27%)^33^, and radiofrequency ablation (40%)^34^, while being comparable to IRE (11%)^9^.

Regarding treatment efficacy, a complete response was observed in 88 patients (75%), a partial response in 21 patients (18%), stable disease in 6 patients (5%), and disease progression in 2 patients (2%). 98% of patients with a Gleason score of 6 or 7a achieved a complete response, while those with PSA levels below 10 ng/ml had a 94% complete response rate and an overall response (OR) rate of 96%. Patients with T1 or T2 stage disease showed a complete response rate of 92% and an overall OR rate of 97%. Significantly lower response rates were observed in patients with more advanced or aggressive disease (T3/T4, N1, or M1 stages, Gleason score > 6, PSA > 10 ng/ml), which is expected, given that 65% of patients in the cohort were classified as T3–T4 stage, indicating locally advanced and nonorgan-confined disease. Furthermore, 28% had a Gleason score of 7b, and 35% had scores of 8 to 10, i.e. high-risk tumors. Additionally, 34% of patients had previously been treated with standard systemic or focal therapies but experienced recurrence or progression.

The preliminary results suggest that ECT yields better outcomes in smaller, organ-confined, and less aggressive tumors, which is consistent with expectations for a local treatment.

As this is a preliminary study, the follow-up period is limited, with a median duration of 9 months (range: 1 to 64 months), preventing the collection of long-term survival data. The 1-year progression-free survival (PFS) rate for the overall cohort was 88% (95% CI: 80%–97%). For patients with early-stage disease (T1 or T2), the 1-year PFS rate was 100%. Similarly, patients with a Gleason score of 6 or 7a had a 96% 1-year PFS rate, while those with a Gleason score of 7b had an 83% 1-year PFS rate (95% CI: 66%–100%). For patients with Gleason scores of 8 or higher, the 1-year PFS rate dropped to 83% (95% CI: 66%–100%). These results are comparable to those from IRE data at 1 year, where recurrence-free survival (RFS) rates are 100% for Gleason 6, 96% for Gleason 7, and 88% for Gleason > 7.^9^

In a subset of the cohort, 10 subjects received ECT while undergoing immunotherapy. No increased toxicity was observed in these patients, and the results in terms of response and progression-free survival were favourable. Only one patient experienced a recurrence, 8.6 months after ECT. This patient was a young individual (55 years old) with high-risk disease, a Gleason score of 9, T4N1M1 staging, and a PSA of 90 ng/ml.

This study has several limitations, including the high variability and heterogeneity in the patients’ disease conditions, the relatively short follow-up duration that precludes comparisons with other studies on recurrence-free survival or overall survival, and the limited data on urinary and erectile function, which should be confirmed in prospective studies with larger, more homogeneous cohorts.

Despite these limitations, the study concludes that ECT is a feasible, safe, and effective treatment for prostate cancer, with extremely low toxicity and side effects. Preliminary results suggest that it offers promising outcomes in terms of local disease control, especially in early-stage tumors, but also in locally advanced cases where other treatments may not be viable. Furthermore, ECT appears to be compatible with immunotherapy without increasing toxicity.
